# Bone loss induced by cancer treatments in breast and prostate cancer patients

**DOI:** 10.1007/s12094-022-02872-1

**Published:** 2022-07-02

**Authors:** Santos Castañeda, Ana Casas, Aránzazu González-del-Alba, Guillermo Martínez-Díaz-Guerra, Xavier Nogués, Cristina Ojeda Thies, Óscar Torregrosa Suau, Álvaro Rodríguez-Lescure

**Affiliations:** 1 Department of Rheumatology, Hospital Universitario de La Princesa, IIS-Princesa, Catedra UAM-Roche, EPID-Future, Universidad Autónoma de Madrid, Madrid, Spain; 2 Department of Medical Oncology, Hospital Virgen del Rocío, Seville, Spain; 3 Department of Medical Oncology, Hospital Universitario Puerta de Hierro-Majadahonda, Madrid, Spain; 4 Department of Endocrinology and Nutrition, Instituto de Investigación imas12, Universidad Complutense, Hospital 12 de Octubre, Madrid, Spain; 5 Department of Internal Medicine, Hospital del Mar, Hospital del Mar Research Institute (IMIM), Centro de Investigación Biomédica en Red de Fragilidad y Envejecimiento Saludable (CIBERFES), Universidad Pompeu Fabra, Barcelona, Spain; 6 Department of Traumatology and Orthopedic Surgery, Hospital Universitario, 12 de Octubre, Madrid, Spain; 7 Department of Internal Medicine, Hospital General Universitario de Elche, Alicante, Spain; 8 Department of Medical Oncology, Hospital General Universitario de Elche, Camino de la Almazara, 11, 03202 Alicante, Spain

**Keywords:** Osteoporosis, Bone health, Cancer, Diagnosis, Bone turnover marker, Fragility fracture, Hormone therapy, Hormone deprivation therapy, Antiresorptive agents

## Abstract

Cancer and cancer therapies are a major factor risk for osteoporosis due to bone loss and deterioration of bone microarchitecture. Both factors contribute to a decrease in bone strength and, consequently, increased bone fragility and risk of fracture. Cancer-associated bone loss is a multifactorial process, and optimal interdisciplinary management of skeletal health, accurate assessment of bone density, and early diagnosis are essential when making decisions aimed at reducing bone loss and fracture risk in patients who have received or are receiving treatment for cancer. In this document, a multidisciplinary group of experts collected the latest evidence on the pathophysiology of osteoporosis and its prevention, diagnosis, and treatment with the support of the Spanish scientific society SEOM. The aim was to provide an up-to-date and in-depth view of osteoporotic risk and its consequences, and to present a series of recommendations aimed at optimizing the management of bone health in the context of cancer.

**Supplementary Information:**

The online version contains supplementary material available at 10.1007/s12094-022-02872-1.

## Introduction

Osteoporosis is defined as a systemic skeletal disease characterized by low bone mass and a deterioration in bone microarchitecture, resulting in an increase in bone fragility and predisposition to fractures [1].

Cancer treatments can have significant negative effects on bone health and cause bone loss or secondary osteoporosis that increases the risk of fractures [2–4]. Moreover, cancer accentuates age-related loss of muscle mass, or sarcopenia, which increases the risk of falls and osteoporotic fractures [5]. This compromises the patient’s functional status, their quality of life, and their very survival.

The aim of this document, drawn up by a group of experts from the Spanish Society of Medical Oncology (SEOM), the Spanish Society for Bone and Mineral Metabolism Research (SEIOMM), the Spanish Society of Rheumatology (SER), and the Spanish Society of Orthopedic Surgery and Traumatology (SECOT) is to provide an up-to-date review of the pathophysiology of the metabolic bone comorbidity osteoporosis in cancer patients. We discuss the biomarkers most widely used in the diagnosis and monitoring of osteoporosis, and the main pharmacological and non-pharmacological measures aimed at preventing and treating bone loss and fractures. We also present advances in surgical and rehabilitation techniques and a series of recommendations based on our clinical experience and expertise to provide a practical up-to-date framework for specialists who routinely monitor these patients.

## Pathophysiology of osteoporosis in cancer patients

The pathophysiology of osteoporosis is multifactorial and varies depending on the underlying disorder. There are multiple risk factors involved in bone loss and fracture (called “osteoporotic, fragility, or low-impact fractures”) (Table 1). Cancer itself and many treatments used in oncology (chemotherapy [CT], radiotherapy [RT], glucocorticoids [GC], or hormone therapies [HT]) are independent risk factors for the development of bone loss, osteoporosis, and fractures [2–4]. There are also other factors that alter bone health, such as prolonged immobilization and/or sedentary lifestyle, primary bone cancer, and bone metastases associated with other types of cancer. Although osteoporosis is a common manifestation in these patients, the pathogenic mechanisms are largely unknown, and most studies focus on patients with breast (BC) and prostate cancer (PC).

**Table 1:** General factors that increase the risk of osteoporosis and fractures

Non-modifiable risk factors	
Age	Personal history of previous fracture
Female sex	Genetic (family history)
Ethnicity (Asian or Caucasian)	Hip fractures in first-degree relatives
Modifiable risk factors	
Low levels of physical activity (prolonged immobilization and/or sedentary lifestyle)	Estrogen deficiency (early menopause, prolonged amenorrhea periods)
Smoking	Low calcium intake or malnutrition
Alcohol consumption (≥ 3 units per day)	Osteoporosis secondary to chronic or consumptive diseases
Low weight (< 58 kg or 127 lb)	Chronic glucocorticoid use
Drugs used in oncology	
*Aromatase inhibitors (BC)*	*Chemotherapy*
Steroidal (exemestane)	Alkylating agents
Non-steroidal (anastrozole, letrozole)	Anthracyclines Docetaxel
*GnRH agonists (BC: goserelin, triptorelin)*	Doxorrubicin
*Selective ER Modulators (BC)*	5-fluorouracil
*Androgen deprivation therapy (PC)*	Other
LHRH analogues (goserelin, buserelin, leuprorelin, triptorelin)	*Other drugs*
LHRH antagonists (goserelin)	Antidepressants and serotonin reuptake inhibitors
Antiandrogens (enzalutamide, bicalutamide, flutamide, nilutamide)	Oral antidiabetics (thiazolidinediones)
Other osteopenizing drugs	
Methotrexate	NSAIDs
Megestrol acetate	Estramustine
Platinum compounds	Ifosfamide
Cyclophosphamide	Radiotherapy
Interferon-alfa	Combination of chemotherapy regimens
Cyclosporine	Valproic acid
Vitamin A	

*ADT* androgen deprivation therapy, *BC* breast cancer, *ER* estrogen receptor, *GnRH* gonadotropin-releasing hormone, *kg* kilograms, *lb* pounds, *LHRH* luteinizing hormone-releasing hormone, *NSAIDs* non-steroidal anti-inflammatory drugs, *PC* prostate cancer

It is important to differentiate between osteoporosis and fractures, because although they share a number of mechanisms and risk factors, others are more specific to each process, such as falls in the case of fractures.

### General risk factors for osteoporosis

**Fig. 1:**
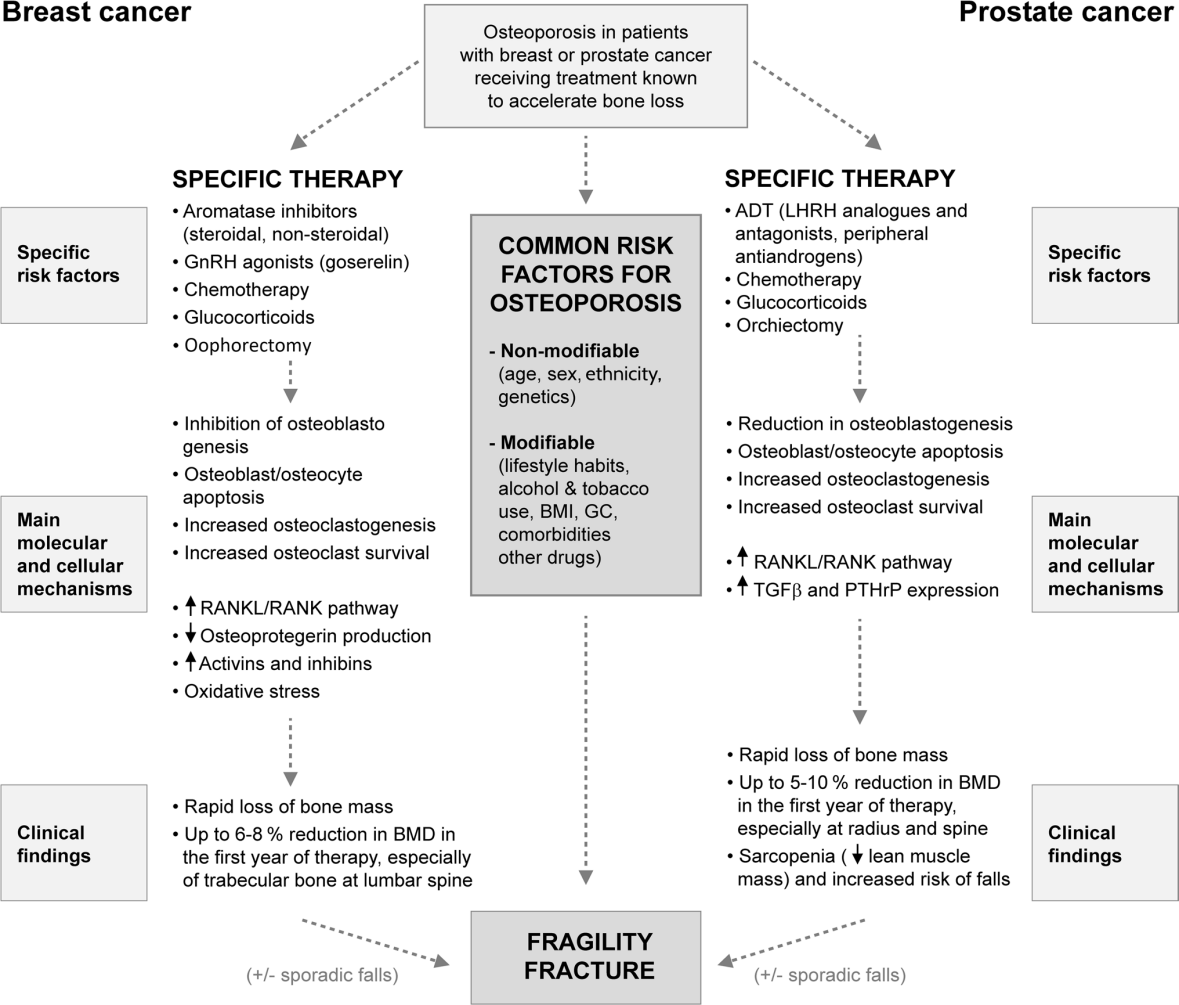
Clinical risk factors and main pathogenic mechanisms of osteoporosis in patients with breast cancer and prostate cancer. *ADT* androgen deprivation therapy, *AI* aromatase inhibitors, BMD bone mineral density, *BMI* body mass index, *GC* glucocorticoids, *LHRH* luteinizing hormone-releasing hormone, *PTHrP* parathyroid hormone-related protein, *RANKL/RANK* receptor activator of the NF-κB (L: ligand), *TGFβ* transforming growth factor beta

Figure 1 summarizes the main risk factors, treatments, and pathogenic mechanisms involved in osteoporosis in cancer patients, particularly in patients with BC and PC.

### Non-modifiable risk factors

The main non-modifiable risk factors are shown in Table 1. Age is one of the main risk factors for both osteoporosis and fracture. In general, the risk of fracture at any location is greater the older the individual [6]. Sex and ethnicity are also important risk factors [6, 7]. Genetics is another fundamental determinant that increases the risk of osteoporosis. In fact, 60%–70% of an individual’s bone mass is estimated to be genetically determined. A history of hip fracture in a first-degree relative doubles the risk of hip fracture in women (relative risk [RR]: 2 [95% confidence interval, CI 1.4–2.9]), regardless of their bone mineral density (BMD) [8]. A history of previous fragility or low-impact fractures is another important determinant of new fractures, with a previous vertebral fracture increasing the risk of hip fracture (RR: 2.5 [95%CI 1.9–3.2]), new vertebral fracture (RR: 1.7 [95%CI 1.4–2]), and proximal humerus fracture (RR: 1.9 [95%CI 1.5–2.4]) [9–12].

### Modifiable risk factors

The presence of associated comorbidities and treatment with GC or other osteopenizing drugs are particularly relevant in the case of cancer patients, who are usually polymedicated (Table 1). The higher the cumulative dose of GC, the higher the risk of osteoporosis. In general, maintaining doses of ≥ 7.5 mg/day, or even ≥ 5 mg/day for more than 3 months, is a risk factor.

Fragility fractures are usually triggered by low-energy trauma that would not ordinarily cause a fracture, such as a fall from standing height or less, and are a sign of underlying osteoporosis or low bone quality. In osteoporosis, the risk of fragility fractures is also related to the individual’s BMD and likelihood of falling, since the fall itself is a fundamental precipitating factor, especially of major non-vertebral fractures (radius, femur, and humerus). In general, the lower the BMD and the higher the number of falls, the greater the risk of a fragility fracture [6, 7, 13, 14].

## Specific risk factors for osteoporosis

### Breast cancer

Osteoporosis associated with BC is mainly linked to estrogen deprivation induced by chemotherapy (CT) and hormone therapy (HT), and more specifically, to the use of non-steroidal aromatase inhibitors (AI) [15, 16]. Estrogens play an essential role in bone homeostasis by binding to estrogen receptors alpha and beta (ERα and ERβ, respectively), which are expressed in both osteoblasts (OB) and osteoclasts (OC), thereby decreasing bone resorption and bone loss [17]. Estrogens promote the proliferation and activity of OBs, decrease the apoptosis of osteocytes (involved in bone formation), and reduce the differentiation and maturation of osteoclastic precursors by increasing the production of osteoprotegerin (OPG) and decreasing the synthesis of osteoclast differentiation and proliferation factor (receptor activator of nuclear factor-κB ligand [RANKL]) [18, 19] (Fig. 1).

Bone loss caused by CT (especially CT involving alkylating agents and/or 5-fluorouracil) or after HT (especially HT using non-steroidal AIs) is rapid, and can reach 6–8% during the first year, especially in trabecular bone. CT-induced ovarian failure has more immediate and difficult-to-reverse effects, while hormone-induced failure can be reversed months after discontinuation, especially in young women. The risk level for osteoporosis in decreasing order is as follows: premenopausal women with CT-induced menopause treated with gonadotropin-releasing hormone (GnRH) agonists; women initially treated with tamoxifen and subsequently treated with AI; and finally, women treated only with AI, particularly those aged < 70 years [14, 20, 21].

Activins and inhibins are other mediators of interest in osteoporosis in cancer patients. Both belong to the transforming growth factor β (TGFβ) superfamily, whose functions are only partially known [18]. Activins are homodimeric peptides secreted by breast tumor cells that inhibit the proliferation of estrogen receptor-positive (ER +) cells. In addition, they stimulate osteoclastogenesis via receptor activator of nuclear factor-κB (RANK), although their effect on bone formation is less well known. Inhibins are heterodimeric proteins also secreted by breast cells that mediate opposite effects in carcinogenesis. At bone level, they cause a bone turnover disorder by inhibiting both osteoblastogenesis and osteoclastogenesis (Fig. 1) [18, 22].

### Prostate cancer

As with estrogen in women, androgens are fundamental in maintaining bone homeostasis in men. Androgens have a double/triple effect on bone tissue. In fact, they increase bone formation and decrease resorption through a direct effect mediated by androgen receptors (AR). Moreover, some androgens are transformed into estrogens at the peripheral level and act through ERα, which is an additional benefit. Thus, low androgen levels are associated with elevated RANKL levels and greater bone resorption [18, 23]. Osteoporosis is present at diagnosis in 25–40% of patients with prostate cancer (PC). Treatment based on surgical or pharmacological hormone deprivation (androgen deprivation therapy [ADT], which includes the use of luteinizing hormone-releasing hormone [LHRH] analogues), reduces testosterone levels to 20% below baseline after 2–4 weeks [18]. This results in rapid losses in BMD (already detectable 6–9 months after the start of treatment), ranging from 5% to 10% in the first year, especially in the radius and spine, which increases the risk of fractures [18, 24, 25]. Fragility fractures appear in up to 20% of patients in the first 5 years of ADT and the risk increases with time and number of doses administered [25, 26]. ADT can increase the risk of osteoporosis from 10%–40% to 80% after 10 years of treatment exposure [18, 27], and 35% of patients experience skeletal fractures. Other factors that enhance this effect are age and low body mass.

Androgens also have a positive effect at muscle level, while the use of ADT causes an increase in total body fat at the expense of a decrease in lean mass [28, 29]. Therefore, ADT produces sarcopenia with rapid loss of muscle mass and increased risk of falls [28, 30].

Other factors that increase the risk of osteoporosis in PC patients are CT (e.g. docetaxel), RT, prolonged use of GC, and interventions such as orchiectomy. In patients with PC treated with 10–12 mg/day prednisone or equivalent for more than 3 months, a 7-to-17-fold increase in the risk of vertebral or hip fracture has been shown [31].

PC may induce osteoporosis independently of hormone treatment, due to an increase in the expression of TGFβ and parathyroid hormone-related protein (PTHrP) [32]. PTHrP increases the growth and survival of prostate tumor cells in vitro (Fig. 1) [33].

Another tumor that can cause osteoporosis through the aforementioned mechanisms is testicular cancer. In this case, the appearance of osteoporosis is related to age and time from orchiectomy.

### Other tumors

Hematology patients undergoing bone marrow transplantation merit special mention. In many cases, these are young women who have received multiple cycles of CT, immunosuppressant and GC before and after transplantation, with frequent gonadal failure, often permanent [34–36]. In patients undergoing allogeneic and autologous transplantation, graft-versus-host-disease and its treatment appear to play an important role in osteoporosis [37]. In general, these patients are at increased risk for osteoporosis, and treatment options often include hormone replacement therapy (HRT) with estrogens.

## Diagnosis and monitoring of osteoporosis in cancer patients

### Role of bone biomarkers in treatment monitoring and evaluation

It is essential to measure bone mass to estimate fracture risk. The main techniques available for quantifying bone mass include dual-energy X-ray absorptiometry (DXA), quantitative computed tomography, and measurements of bone microarchitecture such as the trabecular bone score (TBS). According to the World Health Organization (WHO), DXA is the gold standard for the study of osteoporosis [38].

Bone turnover markers (BTM) are proteins or enzymes secreted by OBs or OCs during the formation or degradation of matrix protein collagen. They are released into the bloodstream, so their detection is useful for the diagnosis and monitoring of osteoporosis, and for the individual assessment of fracture risk [39, 40]. There are two types of BTMs as follows:

- Bone formation markers (BFMs), which derive from formation processes and reflect osteoblastic activity: alkaline phosphatase (ALP), bone-specific alkaline phosphatase (BALP), procollagen type I carboxy-(PICP) and amino-terminal propeptides (PINP), and osteocalcin.
- Bone resorption markers (BRMs), which derive from the resorption processes and reflect osteoclastic activity: cross-linked carboxy- and amino-terminal telopeptides of type I collagen (CTX and NTX), tartrate-resistant acid phosphatase (TRAP), pyridinolines and deoxypyridinolines, hydroxyproline and sialoprotein.

BRMs show variations within the first 3 months of treatment, so they can be used to assess the response to a given antiresorptive therapy. A positive treatment outcome is reflected in a reduction in these markers. At the end of treatment (approximately 3–6 months later), the markers return to their baseline levels. The best BRMs for treatment monitoring are NTX and CTX in urine, and CTX in blood. Response to antiresorptive drugs has been defined as a reduction in BTM levels of more than the least significant change or to a value below the mean levels of the premenopausal reference range [41]. A 25–30% decrease in CTX or PINP has been associated with a reduction in vertebral fracture (nearly a 40%) [42], so this may be a good threshold for use in clinical practice to evaluate the response of patients to antiresorptive therapies. The most widely used BFMs in clinical practice are BALP, osteocalcin and PINP [43].

There are certain considerations with respect to BRMs as follows:

- BRMs measure functional activity and do not quantify bone mass, so they should not be used for the diagnosis of osteoporosis.
- Collagen-dependent BRMs may be altered due to non-bone pathologies, such as chronic liver disease of any origin.
- BALP may be altered depending on the liver ALP values.
- Chronic renal failure may alter the concentration of BRMs.
- Bone fractures may alter BRM values for several months.
- In general, it is preferable to measure BRMs in serum instead of in urine, due to their lower variability.

### Diagnostic and monitoring tests: recommendations and frequency

Assessing the risk of low BMD is important in cancer patients, since they often present premature loss of bone mass that contributes to the risk of osteoporosis, even in the absence of menopause. Recommendations for patient assessment are summarized in Table 2.

**Table 2:** Recommendations for patient diagnostic and monitoring screening

Patient assessment		Comments
Medical history	*Fracture history*	Previous fractures increase the risk of future fractures, regardless of BMD. It is useful to perform a spinal x-ray before starting treatment in order to detect previous asymptomatic fractures. Techniques such as CT, MRI and/or PET can be very useful in determining whether an acute fracture is a bone metastasis
	*Classic risk factors*	Family history of osteoporosis should be included. The FRAX® is an easily reproducible diagnostic tool developed by the University of Sheffield from a meta-analysis of a wide variety of risk factors for osteoporotic fractures (https://www.sheffield.ac.uk/FRAX/). It allows the estimation of the 10-year risk of hip fracture and major osteoporotic fracture, with or without concomitant determination of BMD, although it may underestimate the risk in cancer patients. When using the FRAX® tool in cancer patients, cancer can be considered a “secondary osteoporosis”. One limitation is that this tool does not weigh the number, severity, or location of previous fractures, or the total or cumulative GC treatment
	*Medications*	Treatment review for potentially osteopenizing drugs
	*Fall risk estimation*	Estimation of fall risk
	*Vitamin D*	Vitamin D deficiency is an independent risk factor for low bone mass, falls, and fractures [112]. Determination of 25-hydroxyvitamin D levels allows patients to be classified as normal (> 30 ng/ml), insufficient (20–30 ng/ml) or deficient (< 20 ng/ml)
Physical & complementary examinations	Height	Height should be measured at least once a year and whenever there is suspicion of a new vertebral compression fracture
	BRMs	Variations throughout the day explain why their reproducibility is not a critical factor in the assessment of FR in cancer patients. However, it may be useful to determine BRMs at the beginning of diagnosis or once treatment has started to gain insight into the status of bone metabolism and, above all, to monitor treatment
	BMD	DXA is recommended to measure and compare BMD with previous DXA to assess the progression of osteoporosis. The WHO recommends performing these measurements every 2 years from menopause. The standardized recommendation for menopausal women treated with AI was an annual BMD assessment for the duration of treatment, especially if there is baseline osteopenia or osteoporosis [113]. The ASCO recommends increasing the frequency of DXA follow-up screening if deemed medically necessary based on the results of BMD testing and expected bone loss [84] Fig. 2

*AI* aromatase inhibitors, *ASCO* American Society of Clinical Oncology, *BMD* bone mineral density, *BRMs* bone resorption markers, *CT* computed tomography, *DXA* dual energy X-ray absorptiometry, *FRAX* Fracture Risk Assessment Tool, *GC* glucocorticoid; *FR* fracture risk, *MRI *magnetic resonance imaging, *PET* positron emission tomography, *PMW* postmenopausal women, *PrMW* premenopausal women, *WHO* World Health Organization

**Fig. 2:**
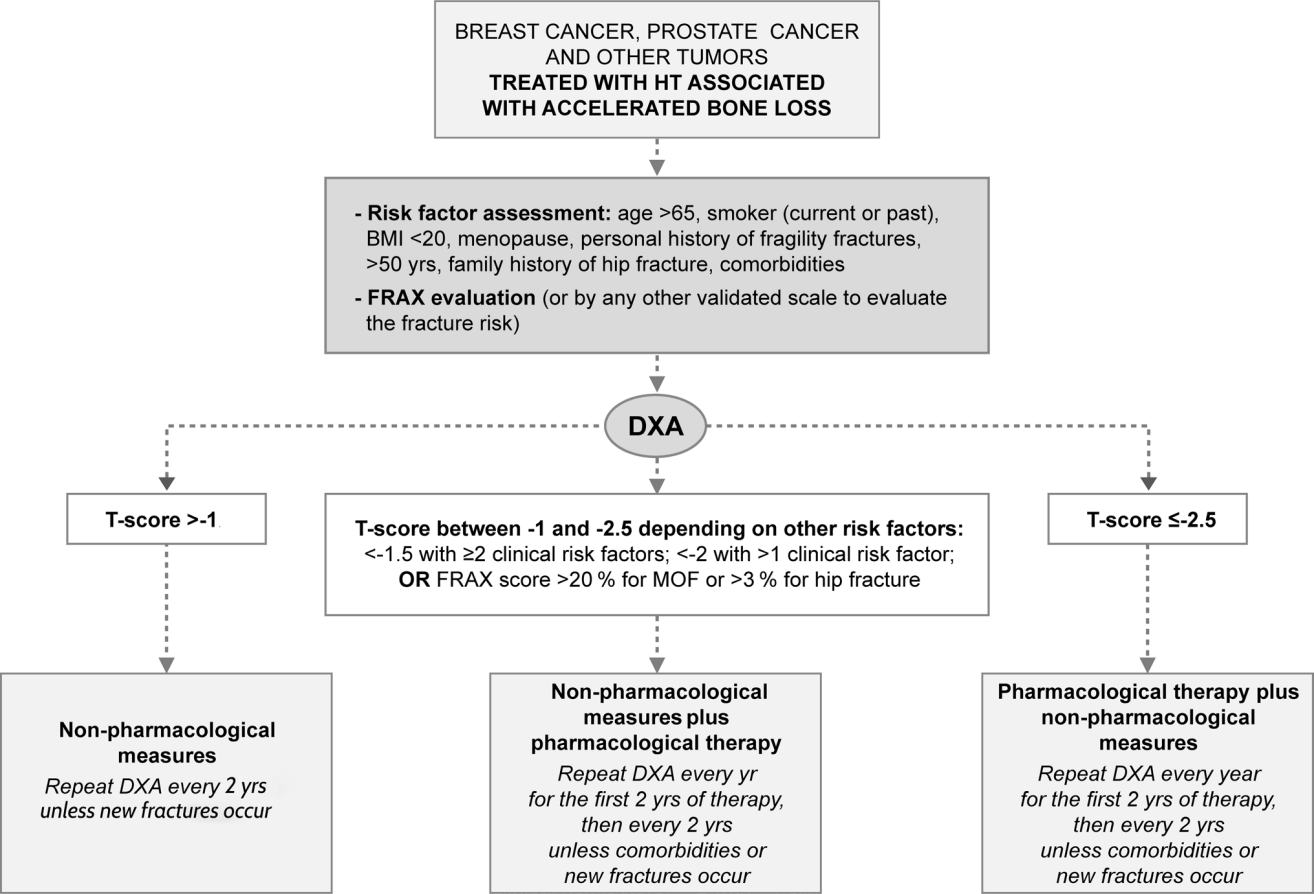
Proposed therapeutic approach to cancer patients with bone loss induced by hormone deprivation therapy. Non-pharmacological measures include the following: regular exercise, calcium 1200 mg/day and vitamin D 800–1000 IU/day or supplements to reach 25(OH)Vit D levels > 50–75 nmol/L (20–30 ng/ml,) if necessary, smoking and alcohol cessation and training to avoid falls. Pharmacological measures are indicated when T-score < -1.5 or < -2 depending on the number of aforementioned clinical risk factors and clinical guideline followed. In addition, dorsolumbar X-ray may be necessary if axial pain appears or a vertebral fracture is suspected. Pharmacological treatment is mandatory in any of the three scenarios mentioned if a prevalent major osteoporotic fracture is confirmed. *DXA* dual energy X-ray absorptiometry, *FRAX* Fracture Risk Assessment Tool, *MOF* major osteoporotic fracture, *yrs* years

### Prevention and treatment of osteoporosis in cancer patients

This section will discuss in detail the main pharmacological and non-pharmacological measures to prevent and treat osteoporosis (Table 3).

**Table 3:** Pharmacological and non-pharmacological measures for the prevention and treatment of osteoporosis in patients with cancer

Non-pharmacological measures	Pharmacological measures
*Smoking cessation*	*Hormone replacement therapies*
*Avoid excess alcohol intake*	*Antiresorptive agents*
*Avoid excess caffeine intake*	Selective estrogen receptor modulators (SERMs)
*Avoid sedentary lifestyle*	Calcitonin
*Prevent falls*	Bisphosphonates (alendronate, risedronate, ibandronate, zoledronate)
*Balanced diet*	Denosumab (anti-RANKL biologic)
*Adequate intake of:*	
Trace minerals	
Proteins	
Vitamin D	
Calcium Combination of calcium and vitamin D	
Physical therapy (improve muscle strength and balance)	

RANKL receptor activator of NF-κB ligand, SERMs selective estrogen receptor modulators

### Non-pharmacological measures

#### Dietary supplements: amino acids, calcium and vitamin D

Age and cancer treatments can impair protein synthesis with decreased response to amino acids and insulin resistance. This is often compounded by the physical or psychological difficulty of achieving adequate intake, a result of the disease itself or the therapeutic sequelae. Adequate protein, calcium, and vitamin D levels are essential for proper bone homeostasis, so it is recommended that they be added to the diet, preferably as dietary supplements because of their greater efficacy and tolerance [44, 45]. Vitamin D plays a fundamental role in calcium and phosphorus homeostasis and is essential to maintain skeletal muscle health, muscle mass and strength, and balance [46]. Vitamin D levels higher than 50 nmol/L (20 ng/mL) are recommended in the general population. Levels above 100 nmol/L (40 ng/mL) have been shown to reduce AI-associated arthralgias [47, 48]. As regards calcium, the necessary dietary intake is estimated to be 1200–1500 mg/day.

All patients starting treatment for osteoporosis should have normal calcium and vitamin D levels at the start of treatment. The National Comprehensive Cancer Network (NCCN) guidelines recommend calcium (1200 mg daily) and vitamin D (800–1000 IU daily) supplements for young women at risk of losing BMD and for women over 50 years of age. For men with PC, calcium (1000 mg daily) and vitamin D (800–1000 IU daily) supplements are recommended from the age of 50 [49, 50]. The European Association of Urology (EAU) mentions the protective role of calcium supplements in patients with PC, of whom 71% receive ADT [51]. Several studies and reviews have confirmed the role of dietary supplements in alleviating the adverse events of ADT on BMD [52]. Monitoring by bone densitometry every 1–2 years (or lengthening the scan interval in the case of stabilization) is also recommended in these patients. BRMs such as serum CTX or urine NTX, or BFMs such as serum PINP every 3–6 months from the start of treatment may be considered.

Obesity and vitamin D deficiency are global health issues. Although evidence from meta-analyses has consistently supported an association between body weight and fat mass and low levels of vitamin D, the underlying pathophysiological mechanisms are complex and many potential confounding factors should be taken into account. Patients with obesity frequently present decreased levels of vitamin D in serum, as this parameter correlates inversely with BMI, body weight or abdominal fat, irrespective of gender, likely leading to an increased risk of osteoporosis [53]. The potential benefit of vitamin D supplementation in obese people has been reported in several studies [54, 55], so the assessment of vitamin D levels in patients with osteoporosis and high BMI is advisable in order to prescribe vitamin D supplementation when necessary.

### Physical exercise

Exercise has been shown to improve a wide spectrum of cancer-related adverse events. Several clinical practice guidelines currently recommend exercise as a key element in the management of cancer patients. The SEOM recently published a position statement on physical exercise and cancer due to its possible impact on the prevention and reduction of complications and relapses [56]. However, there are no specific guidelines for a particular type of exercise, and little is known about the extent to which physical exercise can prevent bone loss.

The results of different randomized trials on the effect of exercise on BMD in cancer survivors have not shown a significant overall improvement in lumbar spine or femoral neck BMD (exercise versus placebo) [57, 58].

In women with BC on treatment, the results of programs combining moderate intensity strength and resistance exercises are inconclusive, largely due to the limitations and heterogeneity of the patient sample and poor adherence to exercise programs [59]. Although not all types of exercise are equally osteogenic, moderate-intensity aerobic exercise has beneficial effects on the lumbar spine BMD in women with BC, and it is also important to encourage adherence to training programs [57, 60–62].

There is still no evidence on the effect of exercise in preventing fractures [63] in men with PC undergoing ADT, although positive effects have been observed on other aspects such as anxiety, bone loss and sexual dysfunction [64].

### Fatty acids

Omega-3 fatty acids have anti-inflammatory potential and are related to PC. It has been suggested that daily use of omega-3 and omega-6 combined with calcium has a positive effect on bone health [65]. However, the results of the various studies analyzed were inconclusive, so larger-scale research is needed to determine the role and effect of these nutrients in the prevention of events, especially fractures.

### Pharmacological measures

Table 3 lists the most used drugs, while the most relevant efficacy and safety clinical trials in patients with BC and PC are presented in Online Resource 1.

### Bisphosphonates: breast cancer

Several randomized clinical trials have shown that bisphosphonates prevent or reduce bone mineral loss in women with BC treated with AI, although they do not significantly reduce the overall incidence of fractures. A systematic review of six studies confirmed with moderate-quality evidence that the RR of skeletal events was not significantly reduced in patients treated with bisphosphonates compared to the placebo or the no bisphosphonate group [66].

The studies with the largest number of patients are the Z-FAST and the ZO-FAST trials (*Zometa-Femara Adjuvant Synergy Trials*). Both evaluated the efficacy of intravenous zoledronic acid (ZOL) in preventing AI-induced bone loss. All patients received calcium (500 mg) and vitamin D (400–800 IU) supplements. In the Z-FAST study, 602 postmenopausal women with early BC who received adjuvant letrozole were randomized to receive upfront or delayed-start intravenous ZOL treatment. At 12 months, BMD was higher in the group that received ZOL up front compared to the delayed-start group, and a significant reduction in NTX and BALP was observed in the first group [67] (Online Resource 1). In the 5-year extension study, a progressive increase in BMD was observed in the upfront treatment group, with significant differences between both groups and no significant differences in the incidence of fractures [68]. One quarter of patients (25%) in the delayed treatment group required ZOL treatment. This suggests that not all women need antiresorptive treatment, and patients should preferably be pre-selected individually on the basis of their fracture risk identified from the BMD and clinical risk factors. In the similarly designed ZO-FAST study, the lumbar spine BMD increased at 36 months in the upfront treatment group, while loss of BMD was observed in the delayed-start group. Twenty-one percent of patients in the delayed group required ZOL treatment during the study [69] (Online Resource 1). Very similar results were obtained in the N03CC trial [70, 71]. It should be noted that the regimen commonly used in non-BC-related menopausal osteoporosis is 5 mg intravenous ZOL annually, which is likely to also be effective in the treatment of AI-induced osteoporosis, although this has not been studied.

With regard to oral bisphosphonates, various trials have assessed their effect on the prevention of AI-induced osteoporosis. The doses studied are the same as those recommended in postmenopausal osteoporosis. Of these, the most extensively studied is risedronate (35 mg/week). In the SABRE trial, 154 postmenopausal patients with BC treated with the AI anastrozole as adjuvant therapy and who had moderate risk of osteoporotic fracture were randomized to receive risedronate or placebo for 2 years. In the risedronate group, the lumbar spine and total hip BMD increased significantly, but decreased in the placebo group [72]. In a more recent placebo-controlled trial that recruited 109 women with low BMD treated with different AIs (anastrozole, letrozole, or exemestane), risedronate achieved greater increases in spine and hip BMD at 24 months compared with placebo [73]. All women received supplemental calcium and vitamin D. Greater reduction in CTX and PINP correlated with a better response in spinal BMD. Risedronate also achieved better conservation of lumbar bone microarchitecture estimated using the TBS [74] (Online Resource 1).

A recent large observational cohort study to evaluate the efficacy of oral bisphosphonates under clinical practice conditions in 36 472 women diagnosed with BC and treated with tamoxifen and/or AI showed that in the subgroup of women treated with AI who had high fracture risk, treatment with oral bisphosphonates reduced the risk of fractures by 30% compared to the group that did not receive bisphosphonates. This is the first real-world study to confirm positive data from previous clinical trials with bisphosphonates, and to report a positive effect on fractures [47].

### Bisphosphonates: prostate cancer

A recent systematic review and meta-analysis of 14 clinical trials has shown the protective effects of bisphosphonates on BMD loss in men with non-metastatic PC receiving ADT, resulting in a significant increase in lumbar, femoral neck and total hip BMD after 12 months of treatment [75] (Table 3). Most of the trials analyzed included administration of intravenous ZOL, alendronate, and to a lesser extent, risedronate. Despite this, it has not been possible to demonstrate a reduction in the fracture risk with oral or intravenous bisphosphonates in these patients, since the number of fractures recorded in the trials was extremely low [76]. For this reason, ASCO clinical guidelines consider oral or intravenous bisphosphonates to be a reasonable option to reduce the fracture risk in patients with non-metastatic PC and ADT [77].

### Adverse events of bisphosphonates

Bisphosphonates are generally well tolerated. In prolonged treatment with intravenous ZOL, the appearance of flu-like symptoms, renal failure, hypocalcemia and osteonecrosis of the jaw (ONJ) should be monitored, among others. ONJ is a very rare problem that is more common in cancer patients with bone metastases who receive monthly doses of intravenous ZOL for long periods. In early BC, the incidence of ONJ is 0.25%. The Z-FAST trial reported two unconfirmed cases of ONJ, and generalized bone pain was most commonly seen in the group randomized to upfront intravenous ZOL versus the delayed-start group [62].

### Denosumab

Denosumab is a human monoclonal antibody directed against RANKL, thus inhibiting the differentiation, proliferation and activity of OCs and, therefore, reducing bone resorption. In clinical trials in postmenopausal women with osteoporosis, denosumab treatment for up to 10 years increased lumbar spine and femoral neck BMD, and reduced the risk of osteoporotic vertebral, hip and non-vertebral fractures [78, 79]. The most common adverse events associated with denosumab are hypocalcemia, diarrhea, eczema and skin infections; the occurrence of hypophosphatemia and ONJ, among other effects, has also been described, albeit relatively less frequently [80].

### Denosumab: breast cancer

In a placebo-controlled clinical trial in women with non-metastatic BC treated with AI and low BMD, the use of denosumab (according to the recommended regimen for postmenopausal osteoporosis) led to a significant increase in lumbar spine, hip and femoral neck BMD and a reduction in BTMs. This effect was independent of the duration of AI therapy. There was no significant effect on the number of fractures [81]. The ABCSG-18 (Adjuvant Denosumab in Breast Cancer Trial) study, which evaluated the protective effect of denosumab against fractures in women with non-metastatic BC on AI, found a 50% reduction in the risk of clinical fracture in women treated with denosumab compared to placebo. In addition, a decrease in the risk of new vertebral fractures and worsening of existing fractures, and a significant increase in lumbar spine, total hip and femoral neck BMD were confirmed. The benefits of denosumab were independent of baseline BMD and age [82] (Table 3).

### Denosumab: prostate cancer

In a trial in patients with PC (> 70 years, or low bone mass with T-score < -1) undergoing surgical castration or ADT with GnRH agonists, a reduction in the risk of new vertebral fractures was observed after 12 months of denosumab treatment (compared to placebo). In addition, a significant and progressive increase in BMD at the lumbar spine, total hip, femoral neck and distal third of the radius was confirmed from the first month of treatment. The incidence of adverse events was similar in both groups, with no cases of ONJ or atypical fractures reported. Cataracts were more frequent in the denosumab group, while the occurrence of bone metastases was more frequent in the placebo group [83] (Online Resource 1).

Different clinical guidelines recommend denosumab as the drug of choice for the prevention of bone loss in patients with non-metastatic PC on ADT [84].

### Other treatments

#### Bone-forming agents

Osteoanabolic agents stimulate the differentiation, function and survival of OBs. These include teriparatide (recombinant form of parathyroid hormone [PTH]), abaloparatide (PTHrP analogue), and romosozumab. After a certain period of time, the conventional treatment for postmenopausal osteoporosis applied in the general population should be administered according to the usual risk factors.

Chronic exposure to PTH or PTHrP analogues causes bone resorption, although intermittent administration has been shown to stimulate bone formation more than resorption in postmenopausal women [85, 86]. Their use in cancer patients on ADT is usually restricted or contraindicated. However, they can be used in patients with osteoporosis with high fracture risk and in cases of ONJ or atypical femoral fracture, both antiresorptive-related complications, as they can facilitate their rapid resolution [87]. Bone-forming treatments are contraindicated in patients with primary or secondary hyperparathyroidism, hypercalcemia, or patients at increased risk of osteosarcoma (such as patients with Paget’s disease of bone and patients who have received RT). A single course of treatment with these drugs is generally recommended for up to 2 years. Although they are usually well tolerated and no associated complications have been described, they can cause hypercalcemia and hypercalciuria to a marginal extent.

Romosozumab (anti-sclerostin monoclonal antibody) is a new anabolic agent approved by the Food and Drug Administration (FDA) in 2019 and the European Medicines Agency (EMA) in 2020, after having demonstrated a reduction in vertebral and non-vertebral fractures compared to placebo and alendronate. Romosozumab is approved for the treatment of severe osteoporosis in postmenopausal women with high fracture risk [88, 89]. There is no formal contraindication for the romosozumab use in cancer patients, despite one of the criteria for patient exclusion in the pivotal clinical trial was a previous history of cancer [90].

### Selective estrogen receptor modulators and calcitonin

Selective estrogen receptor modulators (SERMs) are a group of drugs that have either proestrogenic (mainly cardiovascular, liver, and bone) or antiestrogenic activities (breast, uterus) depending on the target tissue on which they act.

Raloxifene has estrogenic activity in the bone and no proestrogenic activity in the endometrium, unlike other SERMs. It has, therefore, been approved for the prevention and treatment of osteoporosis in postmenopausal women. Tamoxifen, in contrast, has agonist activity at the endometrial level, and has been associated with an increased risk of endometrial cancer. Although not used as a treatment for osteoporosis, tamoxifen contributes to the improvement of bone health in postmenopausal patients who receive it as treatment or prophylaxis against BC [91].

All these drugs carry a slight increase in the risk of thromboembolic events due to their estrogenic agonist activity at the cardiovascular level, as well as climacteric symptoms.

New agents have been added to classic SERMs, such as bazedoxifene, a third-generation SERM that has been approved for the treatment of postmenopausal osteoporosis with increased risk of fractures. The use of these drugs is associated with a lower risk of BC, and they are approved for chemoprevention treatment in patients at high risk of BC.

Calcitonin is a hormone that acts on OCs and inhibits bone resorption. In Spain, it is marketed as salmon calcitonin or eel calcitonin [92]. The former is the most frequently used and has a high affinity for the calcitonin receptor (up to 40 times higher than human calcitonin). Intranasal, oral and parenteral formulations have been developed [93, 94]. Calcitonin has shown a benefit in increasing bone mass in the axial skeleton and in reducing the risk of fracture, although to a lesser extent compared with other agents such as bisphosphonates. Analysis of data from different studies evaluating the safety of prolonged use of calcitonin in the treatment of osteoporosis identified an increase in the incidence of cancer in patients receiving the drug compared to the placebo group (with incidence rates of 0.7–2.4%). Accordingly, EMA issued a statement explaining that the benefits of calcitonin as a treatment for osteoporosis did not outweigh the risk identified in the safety analyses and recommended limiting its indication to acute periods of the disease. The main adverse events associated with administration of this treatment are nausea, vomiting, and hot flushes. Calcitonin is not used as a first-line therapy due to the existence of other drugs that are more effective in preventing bone loss and reducing fracture risk. It is mainly indicated in patients with recent osteoporotic fracture, but should only be administered for 2–5 weeks at the lowest effective dose for the patient, or until resolution of pain. After the acute episode, it is recommended to switch calcitonin to other more effective osteoporosis medications.

### Sequential treatment

Oral bisphosphonates are recommended as first-line treatment of osteoporosis due to their efficacy, safety, accessibility and cost-effectiveness. In patients contraindicated for oral or even intravenous bisphosphonates (as is the case in patients with gastrointestinal disturbances or renal failure) or patients with high fracture risk who have new fracture events despite bisphosphonate treatment (which should be switched to an anabolic agent), other alternatives may be offered based on the fracture risk, efficacy, safety and patient preference. One of these alternatives is denosumab.

The denosumab discontinuation effect has been the subject of recent attention, given the risk of fracture following discontinuation due to a rebound effect on bone resorption observed in clinical series, although this has not been confirmed with high-level evidence. In this respect, a recent systematic review by the European Calcified Tissue Society (ECTS) working group suggests that patients with high fracture risk can maintain denosumab beyond 5 years and even continue treatment for up to 10 years [95]. In case of discontinuation, close patient follow-up or switching to bisphosphonates is recommended, although there is no high-level evidence to date to support the regimen to be followed and its duration.

Finally, romosozumab, a more potent bone-forming agent than PTH, has been shown to be more effective than teriparatide after prolonged treatment with oral bisphosphonates in postmenopausal women, according to the STRUCTURE study [88], although there is still no experience with this drug in cancer patients.

### Osteoporotic fractures in cancer patients: prevention and treatment

The burden of osteoporotic fractures has been growing despite the development of bone-protecting medication, mainly due to population aging. Furthermore, as long-term survival of oncologic disease increases, more patients with osteoporotic fractures will be likely to have a history of cancer, particularly breast cancer [96].

Surgical management of fractures follows a series of principles published by the AO Society (*Arbeitsgemeinschaft für Ostheosynthesefragen*) [97]: (1) restoration of anatomical relationships; (2) fixation providing stability as required by the type of fracture, patient and injury; (3) preservation of blood supply to tissues and bones; and (4) early and safe mobilization of the injured part and the patient as a whole. Several technological innovations in recent decades have helped stabilize osteoporotic fractures while fulfilling these principles, and can be summarized as angular-stable implants, augmentation, and minimally invasive techniques that allow optimal application of biomechanical principles to protect the entire bone [98].

### Surgical management

#### Implants and devices

**Fig. 3:**
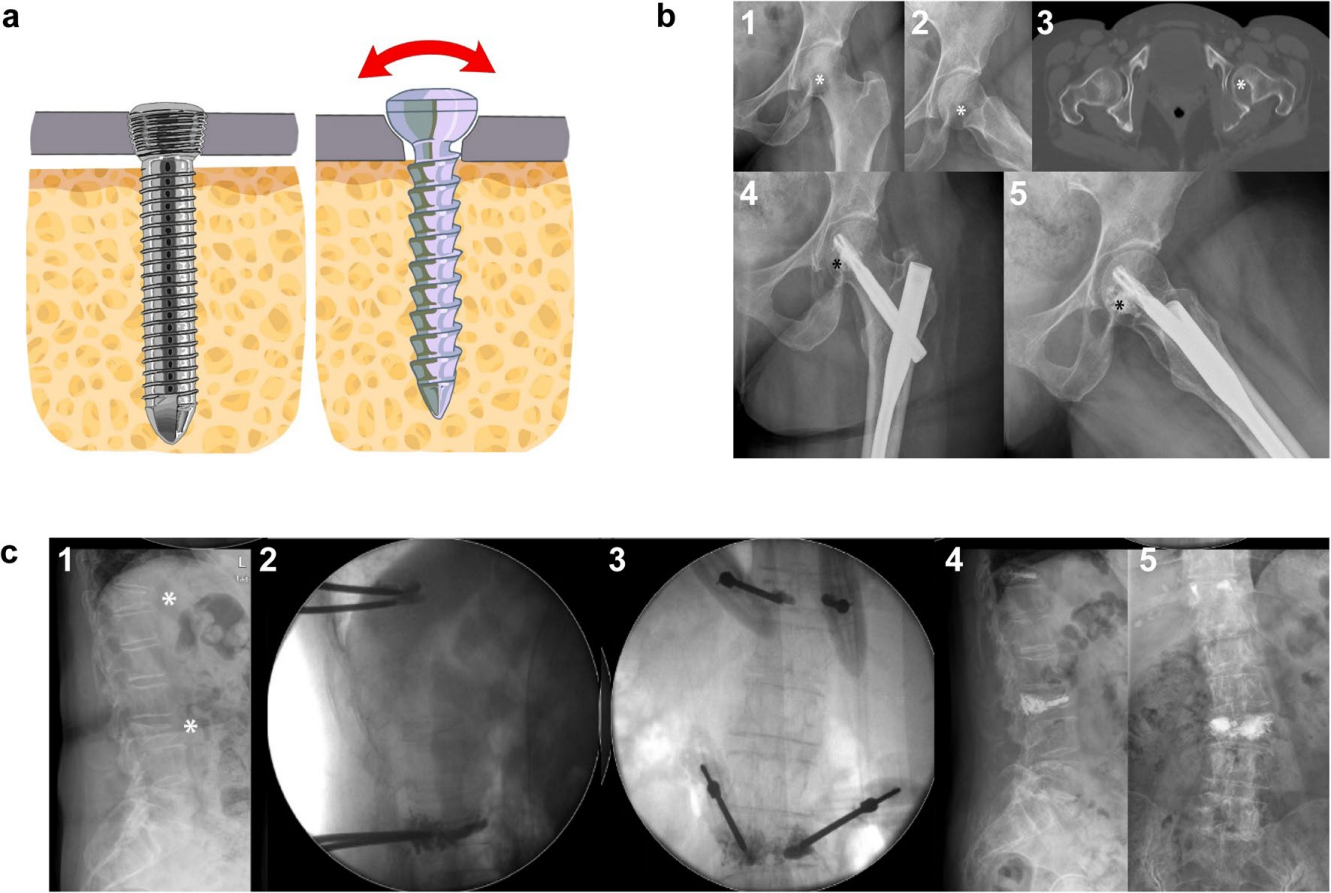
Augmentation technique. **A** Angular stable locking screw and conventional screw. The threaded screw head locks in the plate hole, providing angular stability and reducing shearing (red arrow). This stability reduces the dependence on the bone–plate interface for stability, protecting periosteal tissue; **B** Lytic metastatic lesion (white asterisk) of the postero-inferior aspect of the femoral head in a patient with metastatic renal cancer: [1] AP and [2] axial view in conventional radiographs; [3] axial computed tomography; [4] AP and [5] axial view following internal fixation using a cephalomedullary nail with cement augmentation. Note the filling of the lytic lesion in the femoral head (black asterisk). **C** Fracture of the 11th dorsal and 3rd lumbar vertebra (asterisks) in a patient with multiple myeloma [1]; lateral [2] and anteroposterior [3] intraoperative fluoroscopy of balloon kyphoplasty of the affected vertebrae; lateral [4] and anteroposterior postoperative radiographs [5]. Clinical case courtesy of Dr. Rodrigo Merino, Orthopedic Department, Hospital Universitario 12 de Octubre, Madrid

Angular-stable implants have an additional fixation point between the screw and the implant itself to increase resistance to shearing in fragile or comminuted bone. This added point of fixation is achieved in plates by using locking screw heads that lock into the plate itself through threaded screw heads or locknuts (Fig. 3); in nails, the holes for the locking bolts are threaded or lined with polyethylene or a similar material to lock the screws in place. Locking plates do not rely on bone-to-plate contact for stability, acting as “internal fixators” without excessive periosteal stripping or soft tissue dissection. This allows for indirect reduction and plating using minimally invasive surgery and plate constructs with long working distances that are less stiff and distribute loads across the bone. The trend is towards using longer implants that protect the entire bone (i.e., long cephalomedullary nails), especially in cases with a possibility of metastatic disease [99, 100].

A recent survey of French physicians showed a willingness to use implantable devices to prevent contralateral hip fractures, particularly in oncologic indications [101]. Several techniques have been developed, but experience is limited to small case series and preliminary trials [102], and the cost-effectiveness of these interventions has not been evaluated yet [103].

### Augmentation techniques

Augmentation is the injection of bone cement or bone substitutes in the area of the screws to increase purchase in poor bone and assist load transfer through fenestrations in specially designed cannulated screws (Fig. 2A and B). This technique is also used in vertebral kyphoplasty [104], where bone cement is injected into a cavity in the vertebral body created by balloon expansion (Fig. 2C) to reduce micromotion at the fracture site and increase trabecular bone resistance to compression. Osteoplasty is the application of this technique to bones other than spine and has been used percutaneously in combination with internal fixation for traumatic injuries [105] as well as lytic bone lesions [106].

### Rehabilitation

The fracture fixation construct should be sufficiently stable to enable early mobilization and weight bearing. There is evidence that frail elderly patients are unable to comply with partial weight-bearing [107]; furthermore, early weight-bearing reduces morbidity and mortality [108, 109]. Early intervention has been shown to improve physical function following fracture, particularly hip fractures, though it remains unclear which types of exercise are superior [110].

The effect of exercise interventions seems less marked in patients who have already experienced fractures, although benefits were observed for measures of balance and mobility, fall risk, physical activity, mood, and community outings [111].

## Conclusions

Steady improvements in the effectiveness of cancer treatments have not been accompanied by equally optimal management of skeletal health, which is badly affected by the disease and the treatments themselves. Efforts are needed to raise awareness among physicians and specialists in the care of cancer patients about the importance of monitoring bone health. Many sectors of the healthcare system still remain oblivious to the tremendous impact on quality of life and functional status caused by bone loss from mild fractures and vertebral compression. The healthcare system also plays an essential role in improving understanding among patients of the available treatments, the risks of fracture and bone weakness associated with some therapies, and the diet and lifestyle changes that are most effective in preventing bone loss and fractures. There is still limited knowledge about the risk of osteoporosis associated with a wide range of medical and surgical treatments for cancer. More research is needed to increase the effectiveness and number of available antiresorptive and bone-forming therapies, and more evidence on the effectiveness of combined bone resorption and formation treatments is needed. Other areas, such as the management of severe osteoporosis, early identification of patients with increased or imminent risk of fracture after recent fractures and patient adherence to long-term treatments, also need to be improved. The emergence of new bone-forming therapies and the application of more personalized precision medicine may represent an important advance in the management of bone health in patients with cancer.

Until these issues have been resolved, efforts should be focused on promoting the identification of cancer patients at risk of morbidity due to bone loss and their proper follow-up. In this context, BMD is currently one of the most important tools in the diagnosis and monitoring of these patients.

## Supplementary Information

Below is the link to the electronic supplementary material.

Supplementary file1 (DOCX 65 KB)
