# Correction to: Bone loss induced by cancer treatments in breast and prostate cancer patients

**DOI:** 10.1007/s12094-022-02905-9

**Published:** 2022-08-06

**Authors:** Santos Castañeda, Ana Casas, Aránzazu González-del-Alba, Guillermo Martínez-Díaz-Guerra, Xavier Nogués, Cristina Ojeda Thies, Óscar Torregrosa Suau, Álvaro Rodríguez-Lescure

**Affiliations:** 1 Department of Rheumatology, Hospital Universitario de La Princesa, IIS-Princesa, Catedra UAM-Roche, EPID-Future, Universidad Autónoma de Madrid, Madrid, Spain; 2 Department of Medical Oncology, Hospital Virgen del Rocío, Seville, Spain; 3 Department of Medical Oncology, Hospital Universitario Puerta de Hierro-Majadahonda, Madrid, Spain; 4 Department of Endocrinology and Nutrition, Instituto de Investigación imas12, Universidad Complutense, Hospital 12 de Octubre, Madrid, Spain; 5 Department of Internal Medicine, Hospital del Mar, Hospital del Mar Research Institute (IMIM), Centro de Investigación Biomédica en Red de Fragilidad y Envejecimiento Saludable (CIBERFES), Universidad Pompeu Fabra, Barcelona, Spain; 6 Department of Traumatology and Orthopedic Surgery, Hospital Universitario, 12 de Octubre, Madrid, Spain; 7 Department of Internal Medicine, Hospital General Universitario de Elche, Alicante, Spain; 8 Department of Medical Oncology, Hospital General Universitario de Elche, Camino de la Almazara, 11, 03202 Alicante, Spain

## Correction to: Clinical and Translational Oncology 10.1007/s12094-022-02872-1

In the sentence beginning ‘In this document…’ in the Abstract section of this article, the text ‘In this document, a multidisciplinary group of experts collected the latest evidence on the pathophysiology of osteoporosis and its prevention, diagnosis, and treatment with the support of the Spanish scientific societies SEOM, SER, SEIOMM, and SECOT’ should have read ‘In this document, a multidisciplinary group of experts collected the latest evidence on the pathophysiology of osteoporosis and its prevention, diagnosis, and treatment with the support of the Spanish scientific society SEOM’.

The original article has been corrected.

