# Supplementary material for: Bone loss induced by cancer treatments in breast and prostate cancer patients
Source: Clin Transl Oncol. 2022 Jul 2;24(11):2090–106. doi: 10.1007/s12094-022-02872-1 (PMC9522722; doi:10.1007/s12094-022-02872-1)
Supplement: Supplementary file 1 — Supplementary file1 (DOCX 65 KB) [file 12094_2022_2872_MOESM1_ESM.docx]

**Online Resource 1**

**Clinical and Translational Oncology**

Bone loss induced by cancer treatments in breast and prostate cancer patients

Santos Castañeda^1^, Ana Casas^2^, Aránzazu González-del-Alba^3^, Guillermo Martínez-Díaz-Guerra^4^, Xavier Nogués^5^, Cristina Ojeda Thies^6^, Óscar Torregrosa Suau^7^, Álvaro Rodríguez-Lescure^8*^

^1^ Department of Rheumatology. Hospital Universitario de La Princesa, IIS-Princesa, catedra UAM-Roche, EPID-Future, Universidad Autónoma de Madrid, Madrid, Spain.

^2^ Department of Medical Oncology. Hospital Virgen del Rocío, Seville, Spain.

^3^ Department of Medical Oncology. Hospital Universitario Puerta de Hierro-Majadahonda, Madrid, Spain.

^4^ Department of Endocrinology and Nutrition. Instituto de Investigación imas12, Hospital 12 de Octubre, Universidad Complutense, Madrid, Spain.

^5^ Department of Internal Medicine, Hospital del Mar, Hospital del Mar Research Institute (IMIM), Centro de Investigación Biomédica en Red de Fragilidad y Envejecimiento Saludable (CIBERFES), Universidad Pompeu Fabra, Barcelona, Spain.

^6^ Department of Traumatology and Orthopedic Surgery. Hospital Universitario 12 de Octubre, Madrid, Spain.

^7^ Department of Internal Medicine. Hospital General Universitario de Elche, Alicante, Spain.

^8^ Department of Medical Oncology, Hospital General Universitario de Elche, Alicante, Spain.

***Corresponding author:**

Álvaro Rodríguez-Lescure

Hospital General Universitario de Elche

Camino de la Almazara, 11.

03202 Elche (Spain)

Telephone: +34 696092614

Email: [alescure@geicam.org](mailto:alescure@geicam.org)

**Online resource 1.** Major clinical trials on the efficacy and safety of antiosteoporotic drugs in patients with prostate cancer and breast cancer.

| **Study (Duration)** | **Population** | **Exclusion criteria** | **Randomization** | **Efficacy outcomes: BMD** | **Fractures** | **Safety outcomes** |
| --- | --- | --- | --- | --- | --- | --- |
| Smith et al., 2001 [1]  (1 y.) | Men with advanced or recurrent PC and no bone metastases | Men with Paget's or Cushing's disease, hyperthyroidism, hyperprolactinemia, chronic liver disease, or chronic renal insufficiency, prior therapy with ADT, GC, calcitonin or bisphosphonates | **42 men** were randomly assigned 1:1 to leuprolide (L) or leuprolide plus pamidronate disodium (L+P). All patients received bicalutamide, calcium carbonate and vitamin D | **L arm:** Significant decrease in mean BMD in LS (3.3%), trochanter (2.1%), and TH (1.8%). No changes in FN BMD. Significant loss of trabecular BMD (-8.5%). **L+P arm:** No significant changes |  | AE related to treatment with GnRH agonist (anemia, fatigue, vasomotor flushing) reported in both groups. Serious AEs were reported in 14% and 24% of L and L+P arms, respectively |
| Smith et al., 2003 [2]  (1 y.) | Men with PC beginning ADT (GnRH-agonist w/wo antiandrogen) | Men on calcitonin, bisphosphonates or organ dysfunction or LS BMD T-score <-3 | **156 subjects** randomized 1:1 to receive either ZOL (IV, 4 mg) or placebo every 3 months for 1 y. All subjects were instructed to take calcium/vitamin D supplements | Significant changes in LS BMD: 5.6% increase in ZOL group *vs* 2.2% decrease in placebo group. Significant mean percent change between groups was 7.8% |  | Hot flushes and fatigue in both groups. No significant differences in serious or severe AEs between groups. ZOL did not reduce the ADT efficacy or induce renal dysfunction |
| Ryan et al., 2006 [3]  (1 y.) | Men with non-metastatic PC receiving ADT (GnRH-agonists) | Patients who received previous bisphosphonate therapy, with life expectancy < 1 y., or with FN, TH or LS T-score < -2.5 | **120 patients** randomized 1:1 to receive ZOL (IV) or placebo (P). All patients received calcium/vitamin D supplements | Relative increase in FN, TH and LS BMD was 3.6%, 3.8% and 6.7%, respectively, in ZOL group. Mean BALP and NTX levels decreased. FN, TH and LS BMD decreased in P (≥ 2%). Increased mean BALP and NTX levels |  | Incidence of serious and adverse AEs did not differ between groups. No renal failure or ONJ were reported in ZOL arm |
| Israeli et al., 2007 [4]  (52 weeks) | Patients with non- metastatic PC within 1 y. of starting ADT for an intended duration ≥1 y or who had undergone orchiectomy. All patients were required to have a T-score ≥ –2 in the LS (L2-L4) and TH and life expectancy of ≥1 y | Patients were excluded if they had abnormal renal function or known hypersensitivity to any bisphosphonate, had received previous treatment for osteoporosis within 12 months, or anabolic steroids or growth hormones within 6 months | **222 patients** randomized 1:1 to receive ZOL (IV) or placebo (P). All patients were instructed to take daily calcium/vitamin D supplements | At 52 weeks, BMD increased in the LS (4.7%) and TH (1.6%) in the ZOL arm vs. P. BMD decreased in LS (-2%) and TH (-2.1%). Differences in LS and TH BMD were 6.7% and 3.7%, respectively. ZOL-treated patients with low baseline T-scores had greater LS BMD increases vs. patients with normal baseline T-scores | Trauma related fractures occurred in the ZOL arm (2 patients) and P arm (3 patients) | Incidence and type of AEs were similar between groups. Most common AEs included flu-like illness, fatigue and pyrexia. No ONJ was reported in either group |
| Greenspan et al., 2008 [5]  (2 y.) | Men with non-metastatic PC receiving ADT | Men were excluded if they were receiving medication or had diseases known to affect bone metabolism, had a testosterone not in castrate range, or were previously or currently on a bisphosphonate | **112 patients** randomized 1:1 to receive alendronate (A) or placebo (P) during 1^st^ y. In the 2^nd^ y., A-arm was re-randomized to alendronate (A-A) or placebo (A-P). P-arm was reassigned to alendronate (P-A). All patients received calcium/vitamin D | **A-A group** had the most significant increases in BMD at LS (6.7%), TH (1.6%) and FN (3.2%). Mean BMD increased in **A-P** and **P-A groups** at LS (3.3% and 2.4%). At the one-third distal radius, BMD decreased in all groups except for men who received A for 2 y. At 2 y., BTMs were below baseline | No differences in fracture incidence between groups | No differences in incidence of AEs associated with A, except for myalgia and arthralgia (mostly reported in the groups assigned to A) |
| Smith et al., 2009 [6]  (3 y.) | Men with non-metastatic PT receiving ADT, with low LS, TH, or FN BMD (T-score at < −1.0) at baseline or history of osteoporotic fractures | Concurrently receiving antineoplastic therapy or RT, current/previous exposure to oral bisphosphonates. Patients with a LS, FN or TH BMD T-score < −4.0 or currently receiving treatment for osteoporosis | **1468 patients** randomly assigned 1:1 to receive denosumab (SC) or placebo. All patients were instructed to take daily supplements of calcium/vitamin D | LS BMD increased vs placebo at 2 y. (5.6% vs −1.0%). Denosumab was associated with significant increase in BMD at TH, FN, distal radius and all skeletal sites. At 3 y., PINP and TRAP5b decreased | Denosumab was associated with significant decrease in the incidence of new vertebral fractures | AE rates were similar in both groups. One patient receiving denosumab had hypocalcemia (0.1%) |
| Choo et al., 2013 [7]  (2 y.) | Patients with non-metastatic PC on ADT (GnRH agonists), RT and  LS T score > -2.5 | Nodal or distant metastasis; concurrent/previous cytotoxic medications, pelvic RT contraindicated, ADT-intended duration <20 or >40 months, ADT history for >4 months or >1 month of chronic steroid use | **104 patients** randomized to either risedronate (R) or placebo (P) | At 1 and 2 y. mean LS BMD decreased by 5.77% and 13.55% (P arm) vs. 0.12% and 0.85% (R arm). Percent change for BALP was +60% (P) and 9.54% (R) and +13.8% (P) and +22.3% (R) for CTX |  | The risedronate arm had  more AEs in the GI and pain domain, whereas the placebo had more AEs in the endocrine domain  No ONJ was reported |
| Kachnic et al., 2013 [8]  (3 y.) | Men with non-metastatic PC on LHRH agonist therapy and RT, TH T-scores (< −1.0, > −2.5) |  | **109 patients** randomized 1:1 to ZOL (IV) or observation. All patients received calcium/vitamin D supplements | BMD percent changes were improved with ZOL for LS (6% *vs* −5%), left TH (1% *vs* −8% and left FN (3% *vs* −8%) | Only 1 patient in each arm experienced a  bone fracture | Myocardial ischemia in 1 patient in ZOL arm (possibly treatment-related). ONJ was not reported |
| Klotz et al., 2013 [9]  (1 y.) | Men with non-metastatic PC receiving ADT (leuprolide) | Prior treatment with bisphosphonates, intolerance to oral bisphosphonates or history of metabolic disease | **84 patients** were assigned to oral alendronate (A) and **102** received placebo (P). All subjects took oral daily calcium/vitamin D supplements | Lumbar BMD increased by +1.7% and decreased by 1.9% in A and P group, respectively. Mean BMD percent change between arms was 3.6%. Median TH BMD percent change was +0.7% in A and 1.6% in P. Median NTX and BALP percent change were -3.5% and -2.25% in A-arm and +16.5% and +3.12% in P-arm |  | No significant differences in incidence of AEs between groups. Hypertension was more frequently reported in A group *vs* P arm (2.8% *vs* 0%) |
| Denham et al., 2014 [10] (18 months) | Men with locally advanced non- metastatic PC | Previous treatment with AS or bisphosphonates, previous pelvic RT, osteoporosis resulting in vertebral compression fractures, or abnormal renal function | (**n=1071**) 4 treatment arms in a 2x2 factorial design: short term AS (STAS); STAS + ZOL (IV); intermediate term AS (ITAS) and ITAS +ZOL | TH BMD decreased (STAS, -2.6%; ITAS, -4.1%) at 2 y. FN BMD increased at 2 and 4 y.: STAS+ZOL (0.6% and 1.8%); ITAS+ZOL (0.5% and 1.2%). ZOL prevented AS-induced FN BMD loss | ZOL did not reduce the risk of vertebral and non-vertebral fractures | ONJ was observed in 2 subjects treated with ZOL. Hypocalcemia grade 1 ranged from 2.7% to 8.8% in ZOL treated patients |
| Brufsky et al., 2007 [11]  (1 y.) | Postmenopausal women with ER and/or PR-positive BC and baseline LS and TH T-scores ≥-2.0 | Patients treated with prior letrozole or IV bisphosphonates or with prior fractures were excluded | **602 patients** received oral letrozole and were randomly assigned 1:1 to receive either upfront or delayed ZOL (IV). All subjects were instructed to take daily calcium/vitamin D supplements | Mean significant BMD percent difference between groups was 4.4% for LS and 3.3% for TH. 12.6% of patients with normal baseline BMD in ZOL delayed arm developed mild to moderate osteopenia vs. 3.4% of patients in upfront ZOL arm. Serum NTX and BALP decreased in the upfront ZOL arm and increased in the delayed ZOL |  | Occurrence of AEs was similar between groups except for bone pain, which was higher in the upfront ZOL group (11.3%) vs. 4% in ZOL delayed group. ONJ was not reported in either group |
| Bundred et al., 2008 [12]  (1 y.) | Postmenopausal women with ER-positive EBC and baseline LS and TH T-scores >2.0 | Patients treated with prior letrozole or IV bisphosphonates or with prior fractures were excluded | **1065 patients** received oral letrozole and were randomly assigned 1:1 to receive either upfront or delayed ZOL (IV). All subjects were instructed to take daily calcium/vitamin D supplements | At 1 y., least mean squared percent change in BMD was 5.7% for LS (P < 0.0001) and 6% for TH (P < 0.0001) |  | Occurrence of AE was similar between groups apart from bone pain, which was higher in upfront ZOL group (12.3%) compared to 6.9% of patients in ZOL delayed group, as expected |
| Ellis et al., 2008 [13]  (2 y.) | Women with BC on adjuvant AI treatment. Low BMD (T-score of −1.0 to −2.5). Patients required to have serum vitamin D levels ≥12 ng/mL | Patients with osteoporosis (T-score < −2.5), prior vertebral fracture, current use of bisphosphonates, and use of any antineoplastic therapy (apart from AI) | **252 patients** randomized 1:1 to receive denosumab (D) or placebo (P). All patients were instructed to take calcium/vitamin D supplements | At 1 y., LS BMD increased by 5.5% in D group vs. P group, independently of AI or TX duration. Increase was sustained at 2 y. (7.6% difference between arms) | No vertebral fractures were reported over 24 months | Similar incidence of AEs between groups. Most common AEs included arthralgia, pain in extremity, back pain, and fatigue. No hypocalcemia was reported |
| Hines et al., 2009 [14]  (5 y.) | Postmenopausal women with non-metastatic HR-positive BC initiating AI therapy after TX and evidence of R/M disease | R/M disease, existing LS and/or TH fracture, history of low-intensity fracture, baseline LS or FN BMD T-score < -2.0; any prior treatment with drugs known to affect the skeleton | **558 patients** randomized 1:1 to upfront ZOL (IV) or delayed ZOL. All patients were assigned to take letrozole, vitamin D and calcium | Higher average/percent change in LS, TH and FN BMD, at 1 and 2 y. in upfront ZOL, and lower clinically meaningful BMD loss at LS, FN or TH at 1 y. |  | Significantly higher incidence of limb edema, fatigue, fever, and nausea at 1 y. in upfront ZOL group |
| Eidtmann et al., 2010 [15] (5 y.) | Premenopausal and recently menopausal women with ER-positive and/or PR-positive BC and baseline LS and TH BMD T-score >2.0 |  | **1065 patients** received adjuvant daily letrozole (oral, 2.5 mg/day) and were randomly assigned 1:1 to receive upfront or delayed ZOL | 36 months: Mean percent change from baseline in LS BMD was 4.39% (upfront-ZOL patients) and -4.9% (delayed-ZOL patients). Least-squares mean difference in LS BMD was 5.27%, 7.94% and 9.29% at 1, 2 and 3 y. Mean changes in TH BMD were similar but smaller | Fractures occurred in 24  upfront-ZOL patients and 26 delayed-ZOL patients | Incidence of AEs was similar between groups. Upfront-ZOL patients experienced slightly more flu-like symptoms. Higher number of patients with renal failure/impairment in delayed-ZOL. ONJ was reported in 2 upfront-ZOL patients |
| Van Poznak et al., 2010 [16]  (2 y.) | Postmenopausal women with no metastatic HR–positive EBC (scheduled to receive anastrozole), high risk of fracture with LS or FN T-score <-2.0, prior history of fragility fracture | Exclusion criteria included metastatic disease, recent hormone treatment, and medications known to alter BMD | **234 patients** allocated based on their fracture risk (higher, HR; moderate, MR or lower risk, LR). HR patients (38) received anastrozole+ risedronate (A+R). LR patients (42) received A. MR patients (77) were randomly assigned to receive either A+R or A+ placebo (A+P). LR patients only received A. Calcium/vitamin D were recommended for all patients | **MR patients:** significant increase in LS and TH BMD in A + R vs. A + P (2.2% *v* −1.8%; and 1.8% *v* −1.1%; respectively). **HR patients:** significant increase in LS and TH BMD in A+R group (3.0% and 2.0%, respectively). **LR patients:** significant decrease in LS BMD in A+R arm (−2.1%) and numerical decrease in TH BMD (−0.4%). At 1 y., CTX and PINP decreased (43-46%) in all R groups | No difference in the incidence of fractures between arms | Incidence of AEs was comparable among groups |
| Brufsky et al., 2012 [17]  (5 y.) | Postmenopausal women with a history of surgically resectable ER and/or PR-positive EBC receiving letrozole |  | **602 patients** randomized 1:1 to receive upfront or delayed-start ZOL (IV) | Least square means of LS and FN BMD were 8.9% and 6.7% in upfront ZOL and delayed ZOL arms, respectively (P < .0001).  Least squares mean BMD difference between groups steadily increased from baseline to month 61 for LS (4.3% to 8.9%) and TH (3.2% to 6.7%) | The incidence of fractures was 9.3% in delayed ZOL group vs 11% in upfront ZOL | Types and rates of AE were similar between groups |
| Rhee et al., 2013 [18]  (6 months) | Postmenopausal women with HR-positive EBC on AI treatment (anastrozole or letrozole) | Clinical and/or radiological evidence of distant metastasis, previous use of  bisphosphonates or contraindication for bisphosphonates or use of drugs affecting bone metabolism | **98 patients** randomized 1:1 to receive alendronate combined with calcitriol (AC) or placebo (P). All patients received daily calcium/vitamin D supplements | In P group, LS BMD decreased by 3.5% after AI initiation while it was well maintained in AC group. LS BMD difference was ~3.0% between groups. TH BMD decreased by 1.3% in P vs 0.5% in AC. Serum CTX was markedly higher in P group after AI initiation. Change difference in CTX was 72.4%, between AC and P arms | No differences in the risk of major osteoporotic fractures or TH fractures at 10 y. | No significant differences in incidence of AEs between groups  . |
| Sestak et al., 2014 [19]  (5 y.) | Healthy postmenopausal  women at increased risk of BC on anastrozole (Study IBIS-II) | Premenopausal status, previous diagnosis of invasive cancers, current use of selective ER modulators for ≥6 months or evidence of severe osteoporosis (T-score <–4·0) | **260 women** in stratum II were randomly assigned to receive risedronate (R; n=137) or matched placebo (P; n=123).  Calcium/vitamin D were recommended for all patients | 36 months: Women in stratum II randomly allocated to A+R achieved a mean LS BMD increase of 1.1% vs. -2.6% in P. TH BMD decreased by -0.7% for A+R vs. 3.5% decrease in A+P arm (p=0·000). 12 months: NTX/Creatinine decreased by 13% in R vs. 11.7% in P | The incidence rate for fractures in both arms was similar | Incidence of AEs was similar between groups within each stratum. In R arm, no serious AEs, such as ONJ or serious GI problems were reported after 3 y. of follow-up |
| Greenspan et al., 2015 [20]  (2 y.) | Postmenopausal women with HR-positive BC, > 55 y., currently receiving AI (including anastrozole, letrozole, or exemestane) | Women treated with a bisphosphonate in the previous year or on any medication known to affect bone and mineral metabolism such as GC | **109 patients** randomly assigned 1:1 to receive oral risedronate (R) or placebo (P) | LS BMD increased in R group and decreased in P arm over 1 and 2 y. The adjusted difference was 3.9% in favor of R group at 2 y. At 1 and 2 y., TH BMD increased more in R than in P. The adjusted difference at 2 y. was 3.2% in favor of R arm. Similar differences were achieved at FN with 2.6% of adjusted difference at 2 y. In R, CTX and PINP decreased at 1 and 2 y. |  | 94% had no serious AEs. There were no differences between the groups |
| Gnant et al., 2015 [21]  (3 y.) | Postmenopausal women with non-metastatic ER-positive or PR-positive BC receiving adjuvant non-steroidal AI | Prior or concomitant treatment with Tx or SERM. Prior treatment with bisphosphonates | **3420 patients** randomly assigned 1:1 to receive either denosumab (D) or placebo (P). All patients received calcium/vitamin D | At 3 y., D arm had a significant relative increase in LS, TH and FN BMD (10.02%, 7.92%, and 6.51%, respectively). 10%, 17% and 22% of D patients lost LS, TH or FN BMD vs. 74%, 78% and 75% of P patients | Time to first clinical fracture  was delayed in D arm. 5% (D arm) vs.9.6% patients (P group) had fractures | Total incidence of AEs did not differ between D or P arms (80% vs. 79%) nor did the incidence of serious AE (30% of each group). Main AEs were arthralgia and other AI-related symptoms |
| Lester et al., 2008 [22]  (2 y.) | Postmenopausal women with ER-positive BC surgically treated | Patients were excluded if menopause was induced by either prior CT or drug therapy, were receiving medication with effects on bone, had abnormal renal function or disorders of bone metabolism or previous bilateral hip fractures | All patients received anastrozole and calcium/vitamin D supplements. **50 osteopenic patients** were randomized 1:1 to receive ibandronate (I) or placebo (P) | I-patients gained +2.98% and +0.60% at LS and TH BMD, respectively. In the P arm, mean BMD declines were observed at 1 and 2 y. at LS (-2.35% and -3.22%) and TH (-2.27% and -3.90), at both sites, at each time point. At 1-y., mean percentage change from baseline was -30.9%,  -26.3%, and -22.8% for uNTX, sCTX, and sBALP, respectively in I arm (all highly significant) | No fragility fractures were reported in either group | Upper GI tract symptoms such as nausea and indigestion were experienced by 16% of patients in I arm vs. 0% in P group |

ADT androgen deprivation therapy, AE adverse events, AI aromatase inhibitors, AS androgen suppression, BC/EBC breast cancer/early breast cancer, BM bone mass, BMD bone mineral density, BALP bone-specific alkaline phosphatase, BTM bone turnover markers, Cr creatinine, ER estrogen receptor, FN femoral neck, GC glucocorticoids, GI gastrointestinal, GnRH gonadotropin-releasing hormone, HR hormone receptor, IV intravenous, LHRH luteinizing hormone-releasing hormone, LS lumbar spine, ONJ osteonecrosis of the jaw, PC prostate cancer, PINP procollagen type I N-terminal propeptide, PR progesterone receptor, PTH parathyroid hormone, PTHrP parathyroid hormone-related protein, RANKL/RANK receptor activator of the NF-κB (L: ligand), SC subcutaneous, SERMs selective estrogen receptor modulators, R/M recurrent/metastatic, RT radiotherapy, TGFβ transforming growth factor beta, TH total hip, TRAP5b tartrate-resistant acid phosphatase 5b, Tx tamoxifen, w/wo with/without, y year(s), ZOL zoledronic acid /zoledronate.

**References**

1. Smith, M.R., McGovern, F.J., Zietman, A.L., Fallon, M.A., Hayden, D.L., Schoenfeld, D.A., Kantoff, P.W., and Finkelstein, J.S., Pamidronate to prevent bone loss during androgen-deprivation therapy for prostate cancer. N Engl J Med, 2001. **345**(13): p. 948-55.

2. Smith, M.R., Eastham, J., Gleason, D.M., Shasha, D., Tchekmedyian, S., and Zinner, N., Randomized controlled trial of zoledronic acid to prevent bone loss in men receiving androgen deprivation therapy for nonmetastatic prostate cancer. J Urol, 2003. **169**(6): p. 2008-12.

3. Ryan, C.W., Huo, D., Demers, L.M., Beer, T.M., and Lacerna, L.V., Zoledronic acid initiated during the first year of androgen deprivation therapy increases bone mineral density in patients with prostate cancer. J Urol, 2006. **176**(3): p. 972-8; discussion 978.

4. Israeli, R.S., Rosenberg, S.J., Saltzstein, D.R., Gottesman, J.E., Goldstein, H.R., Hull, G.W., Tran, D.N., Warsi, G.M., and Lacerna, L.V., The effect of zoledronic acid on bone mineral density in patients undergoing androgen deprivation therapy. Clin Genitourin Cancer, 2007. **5**(4): p. 271-7.

5. Greenspan, S.L., Nelson, J.B., Trump, D.L., Wagner, J.M., Miller, M.E., Perera, S., and Resnick, N.M., Skeletal health after continuation, withdrawal, or delay of alendronate in men with prostate cancer undergoing androgen-deprivation therapy. J Clin Oncol, 2008. **26**(27): p. 4426-34.

6. Smith, M.R., Egerdie, B., Hernandez Toriz, N., Feldman, R., Tammela, T.L., Saad, F., Heracek, J., Szwedowski, M., Ke, C., Kupic, A., Leder, B.Z., Goessl, C., and Denosumab, H.P.C.S.G., Denosumab in men receiving androgen-deprivation therapy for prostate cancer. N Engl J Med, 2009. **361**(8): p. 745-55.

7. Choo, R., Lukka, H., Cheung, P., Corbett, T., Briones-Urbina, R., Vieth, R., Ehrlich, L., Kiss, A., and Danjoux, C., Randomized, double-blinded, placebo-controlled, trial of risedronate for the prevention of bone mineral density loss in nonmetastatic prostate cancer patients receiving radiation therapy plus androgen deprivation therapy. Int J Radiat Oncol Biol Phys, 2013. **85**(5): p. 1239-45.

8. Kachnic, L.A., Pugh, S.L., Tai, P., Smith, M., Gore, E., Shah, A.B., Martin, A.G., Kim, H.E., Nabid, A., and Lawton, C.A., RTOG 0518: randomized phase III trial to evaluate zoledronic acid for prevention of osteoporosis and associated fractures in prostate cancer patients. Prostate Cancer Prostatic Dis, 2013. **16**(4): p. 382-6.

9. Klotz, L.H., McNeill, I.Y., Kebabdjian, M., Zhang, L., Chin, J.L., and Canadian Urology Research, C., A phase 3, double-blind, randomised, parallel-group, placebo-controlled study of oral weekly alendronate for the prevention of androgen deprivation bone loss in nonmetastatic prostate cancer: the Cancer and Osteoporosis Research with Alendronate and Leuprolide (CORAL) study. Eur Urol, 2013. **63**(5): p. 927-35.

10. Denham, J.W., Nowitz, M., Joseph, D., Duchesne, G., Spry, N.A., Lamb, D.S., Matthews, J., Turner, S., Atkinson, C., Tai, K.H., Gogna, N.K., Kenny, L., Diamond, T., Smart, R., Rowan, D., Moscato, P., Vimieiro, R., Woodfield, R., Lynch, K., Delahunt, B., Murray, J., D'Este, C., McElduff, P., Steigler, A., Kautto, A., and Ball, J., Impact of androgen suppression and zoledronic acid on bone mineral density and fractures in the Trans-Tasman Radiation Oncology Group (TROG) 03.04 Randomised Androgen Deprivation and Radiotherapy (RADAR) randomized controlled trial for locally advanced prostate cancer. BJU Int, 2014. **114**(3): p. 344-53.

11. Brufsky, A., Harker, W.G., Beck, J.T., Carroll, R., Tan-Chiu, E., Seidler, C., Hohneker, J., Lacerna, L., Petrone, S., and Perez, E.A., Zoledronic acid inhibits adjuvant letrozole-induced bone loss in postmenopausal women with early breast cancer. J Clin Oncol, 2007. **25**(7): p. 829-36.

12. Bundred, N.J., Campbell, I.D., Davidson, N., DeBoer, R.H., Eidtmann, H., Monnier, A., Neven, P., von Minckwitz, G., Miller, J.C., Schenk, N.L., and Coleman, R.E., Effective inhibition of aromatase inhibitor-associated bone loss by zoledronic acid in postmenopausal women with early breast cancer receiving adjuvant letrozole: ZO-FAST Study results. Cancer, 2008. **112**(5): p. 1001-10.

13. Ellis, G.K., Bone, H.G., Chlebowski, R., Paul, D., Spadafora, S., Smith, J., Fan, M., and Jun, S., Randomized trial of denosumab in patients receiving adjuvant aromatase inhibitors for nonmetastatic breast cancer. J Clin Oncol, 2008. **26**(30): p. 4875-82.

14. Hines, S.L., Mincey, B., Dentchev, T., Sloan, J.A., Perez, E.A., Johnson, D.B., Schaefer, P.L., Alberts, S., Liu, H., Kahanic, S., Mazurczak, M.A., Nikcevich, D.A., and Loprinzi, C.L., Immediate versus delayed zoledronic acid for prevention of bone loss in postmenopausal women with breast cancer starting letrozole after tamoxifen-N03CC. Breast Cancer Res Treat, 2009. **117**(3): p. 603-9.

15. Eidtmann, H., de Boer, R., Bundred, N., Llombart-Cussac, A., Davidson, N., Neven, P., von Minckwitz, G., Miller, J., Schenk, N., and Coleman, R., Efficacy of zoledronic acid in postmenopausal women with early breast cancer receiving adjuvant letrozole: 36-month results of the ZO-FAST Study. Ann Oncol, 2010. **21**(11): p. 2188-2194.

16. Van Poznak, C., Hannon, R.A., Mackey, J.R., Campone, M., Apffelstaedt, J.P., Clack, G., Barlow, D., Makris, A., and Eastell, R., Prevention of aromatase inhibitor-induced bone loss using risedronate: the SABRE trial. J Clin Oncol, 2010. **28**(6): p. 967-75.

17. Brufsky, A.M., Harker, W.G., Beck, J.T., Bosserman, L., Vogel, C., Seidler, C., Jin, L., Warsi, G., Argonza-Aviles, E., Hohneker, J., Ericson, S.G., and Perez, E.A., Final 5-year results of Z-FAST trial: adjuvant zoledronic acid maintains bone mass in postmenopausal breast cancer patients receiving letrozole. Cancer, 2012. **118**(5): p. 1192-201.

18. Rhee, Y., Song, K., Park, S., Park, H.S., Lim, S.K., and Park, B.W., Efficacy of a combined alendronate and calcitriol agent (Maxmarvil(R)) in Korean postmenopausal women with early breast cancer receiving aromatase inhibitor: a double-blind, randomized, placebo-controlled study. Endocr J, 2013. **60**(2): p. 167-72.

19. Sestak, I., Singh, S., Cuzick, J., Blake, G.M., Patel, R., Gossiel, F., Coleman, R., Dowsett, M., Forbes, J.F., Howell, A., and Eastell, R., Changes in bone mineral density at 3 years in postmenopausal women receiving anastrozole and risedronate in the IBIS-II bone substudy: an international, double-blind, randomised, placebo-controlled trial. Lancet Oncol, 2014. **15**(13): p. 1460-1468.

20. Greenspan, S.L., Vujevich, K.T., Brufsky, A., Lembersky, B.C., van Londen, G.J., Jankowitz, R.C., Puhalla, S.L., Rastogi, P., and Perera, S., Prevention of bone loss with risedronate in breast cancer survivors: a randomized, controlled clinical trial. Osteoporos Int, 2015. **26**(6): p. 1857-64.

21. Gnant, M., Pfeiler, G., Dubsky, P.C., Hubalek, M., Greil, R., Jakesz, R., Wette, V., Balic, M., Haslbauer, F., Melbinger, E., Bjelic-Radisic, V., Artner-Matuschek, S., Fitzal, F., Marth, C., Sevelda, P., Mlineritsch, B., Steger, G.G., Manfreda, D., Exner, R., Egle, D., Bergh, J., Kainberger, F., Talbot, S., Warner, D., Fesl, C., Singer, C.F., Austrian, B., and Colorectal Cancer Study, G., Adjuvant denosumab in breast cancer (ABCSG-18): a multicentre, randomised, double-blind, placebo-controlled trial. Lancet, 2015. **386**(9992): p. 433-43.

22. Lester, J.E., Dodwell, D., Purohit, O.P., Gutcher, S.A., Ellis, S.P., Thorpe, R., Horsman, J.M., Brown, J.E., Hannon, R.A., and Coleman, R.E., Prevention of anastrozole-induced bone loss with monthly oral ibandronate during adjuvant aromatase inhibitor therapy for breast cancer. Clin Cancer Res, 2008. **14**(19): p. 6336-42.
